# Molecular imaging of a fluorescent antibody against epidermal growth factor receptor detects high-grade glioma

**DOI:** 10.1038/s41598-021-84831-4

**Published:** 2021-03-11

**Authors:** Quan Zhou, Johana C. M. Vega Leonel, Michelle Rai Santoso, Christy Wilson, Nynke S. van den Berg, Carmel T. Chan, Muna Aryal, Hannes Vogel, Romain Cayrol, Michael J. Mandella, Frank Schonig, Guolan Lu, Sanjiv S. Gambhir, Michael E. Moseley, Eben L. Rosenthal, Gerald A. Grant

**Affiliations:** 1 Department of Neurosurgery, Stanford University School of Medicine, Stanford, CA 94305 USA; 2 Department of Otolaryngology-Head and Neck Surgery, Stanford University School of Medicine, Stanford, CA USA; 3 Department of Cardiovascular Medicine, Stanford University School of Medicine, Stanford, CA USA; 4 Department of Radiology, Stanford University School of Medicine, Stanford, CA USA; 5 Department of Pathology, Stanford University, Stanford, CA USA; 6 Institute of Quantitative Health Science and Engineering, Michigan State University, Lansing, MI USA; 7 Stanford Health Care, Stanford Medical Center, Stanford, CA USA

**Keywords:** CNS cancer, Tumour biomarkers, Cancer, Medical research, Molecular medicine, Neurological disorders

## Abstract

The prognosis for high-grade glioma (HGG) remains dismal and the extent of resection correlates with overall survival and progression free disease. Epidermal growth factor receptor (EGFR) is a biomarker heterogeneously expressed in HGG. We assessed the feasibility of detecting HGG using near-infrared fluorescent antibody targeting EGFR. Mice bearing orthotopic HGG xenografts with modest EGFR expression were imaged in vivo after systemic panitumumab-IRDye800 injection to assess its tumor-specific uptake macroscopically over 14 days, and microscopically ex vivo. EGFR immunohistochemical staining of 59 tumor specimens from 35 HGG patients was scored by pathologists and expression levels were compared to that of mouse xenografts. Intratumoral distribution of panitumumab-IRDye800 correlated with near-infrared fluorescence and EGFR expression. Fluorescence distinguished tumor cells with 90% specificity and 82.5% sensitivity. Target-to-background ratios peaked at 14 h post panitumumab-IRDye800 infusion, reaching 19.5 in vivo and 7.6 ex vivo, respectively. Equivalent or higher EGFR protein expression compared to the mouse xenografts was present in 77.1% HGG patients. Age, combined with IDH-wildtype cerebral tumor, was predictive of greater EGFR protein expression in human tumors. Tumor specific uptake of panitumumab-IRDye800 provided remarkable contrast and a flexible imaging window for fluorescence-guided identification of HGGs despite modest EGFR expression.

## Introduction

High-grade gliomas (HGGs) are the most common primary malignant brain tumors in adults and the leading cause of cancer-related deaths in children, carrying a poor prognosis despite intensive treatments with surgery, radiotherapy, and chemotherapy^1,2^. As extent of resection correlates with overall survival and progression free disease^3–5^, fluorescence guided surgery that targets specific HGG biomarkers can enhance intraoperative tumor visualization and delineation of tumor margins and improve overall survival of patients.

Epidermal growth factor receptor (EGFR) is an attractive biomarker for HGG imaging. Significantly higher EGFR protein overexpression and gene amplification are more characteristic of high-grade gliomas compared to low-grade gliomas, and are implicated in tumor cell migration and aggressiveness^6,7^. In a fluorescence imaging study, the IRDye800CW (Ex/Em: 774/789 nm) labeled therapeutic antibody, panitumumab-IRDye800, bound to EGFR positive rat glioma cells with higher affinity than the fluorescent EGFR ligand, EGF800^8^. Moreover, this fully humanized EGFR antibody has an improved safety profile compared to its chimeric counterpart, cetuximab^9,10^.

A major barrier to the clinical translation of EGFR targeting imaging probes is the heterogeneity of EGFR protein expression which can vary by orders of magnitude in human HGGs^11,12^. Despite a widely recognized problem in cancer research, animal imaging studies often adopt subcutaneous xenograft models with exceptionally high target expressions that poorly represent actual malignancies in the brain^12,13^. This study examined the feasibility of detecting human HGGs via fluorescence imaging using panitumumab-IRDye800 in a preclinical orthotopic tumor model with only modest level of EGFR expression which was benchmarked against similar expression levels in patient HGG tissues. Since biopsies are not usually taken before resection surgeries, HGG specimens were characterized to stratify a patient population with positive EGFR protein expression that would benefit most from intraoperative targeted imaging to assist in resection.

## Results

### Panitumumab-IRDye800 detects EGFR protein expressed in human glioma cells in vitro

**Figure 1:**
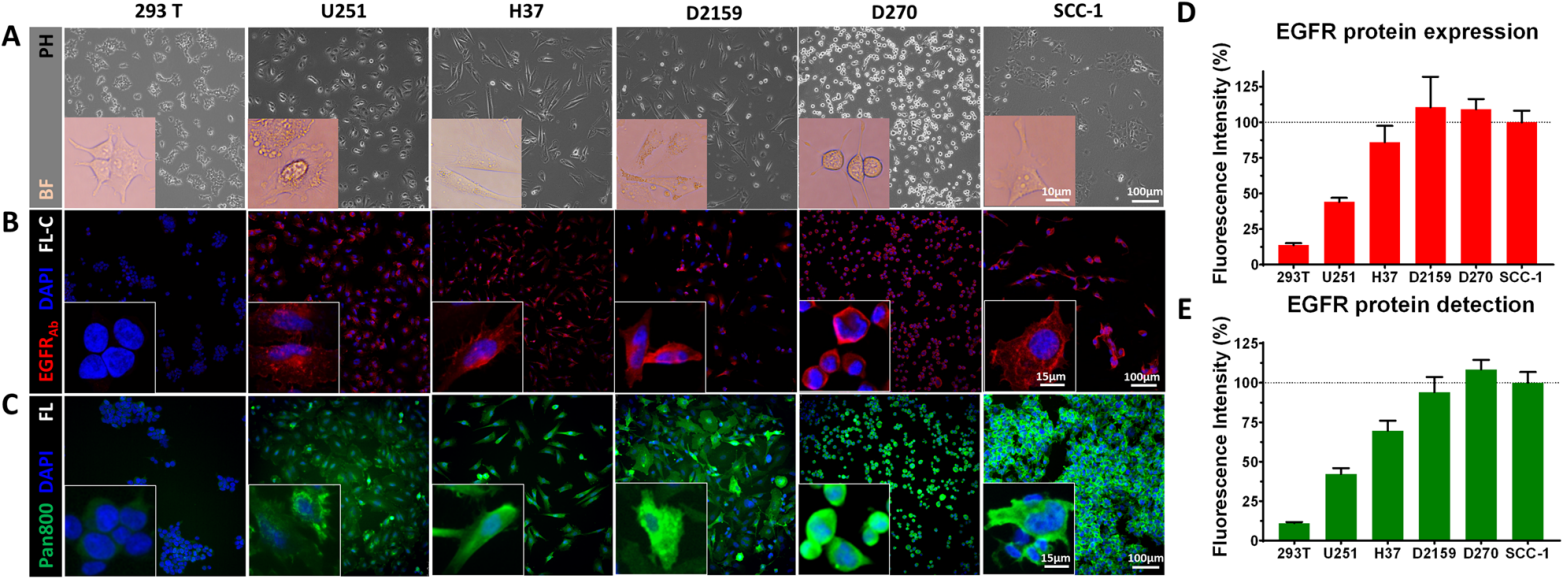
Panitumumab-IRDye800 specifically detects EGFR protein expression in human malignant glioma cells. (**A**) Phase contrast (PH) microscopy images confirmed the healthy growth and expected morphology of six cell lines in vitro before EGFR immunostaining, including human xenograft glioma cell lines (D270, H37, D2159 and U251), positive control (SCC-1) and negative control (293 T). *Insets*: bright field (BF) microscopic images of individual cell morphology. Fixed cells were immunostained with (**B**) a commercial EGFR antibody and (**C**) panitumumab-IRDye800 (pan800). *Insets*: high magnification views of plasma membrane staining on individual cells. EGFR_Ab_: commercial epidermal growth factor receptor antibody; DAPI: 4′,6-diamidino-2-phenylindole (nuclear stain); *FL-C* fluorescence confocal microscopy. *FL* fluorescence microscopy. (**D**) Mean fluorescence intensity, with standard error of the mean (*n* = 10), of rabbit-anti-human EGFR antibody and (**E**) panitumumab-IRDye800, normalized to SCC-1 (as 100%, *dotted lines*).

Four human glioma cell lines, U251, H37, D2159 and D270, Fig. 1A, expressed EGFR protein probed by a commercial EGFR antibody with immunofluorescence assay, Fig. 1B. NIR fluorescent labeled EGFR antibody, panitumumab-IRDye800, detected EGFR protein expression in the same cell lines, Fig. 1C. EGFR protein was found on the cytoplasmic membrane of individual cells (Fig. 1B,C *insets*) with 293 T as negative control. Cell surface EGFR expression normalized to positive control (SCC-1) was lowest in U251 cells (MFI = 44.1 ± 2.8%) among the four lines tested, Fig. 1D. Panitumumab-IRDye800 exhibited EGFR specificity and a similar level of fluorescence in U251 cells (MFI = 42.2 ± 3.6%) in immunofluorescence assays, Fig. 1E, and demonstrated high in vitro affinity (IC50 = 43.6 nM) in a competition assay, Supplementary Fig. S1. U251 was selected to establish the orthotopic brain tumor model in mice due to its modest EGFR expression in tumors and to test the feasibility of detecting HGG with panitumumab-IRDye800 in vivo.

### U251 produces contrast enhancing orthotopic HGG xenograft tumor in mice

**Figure 2:**
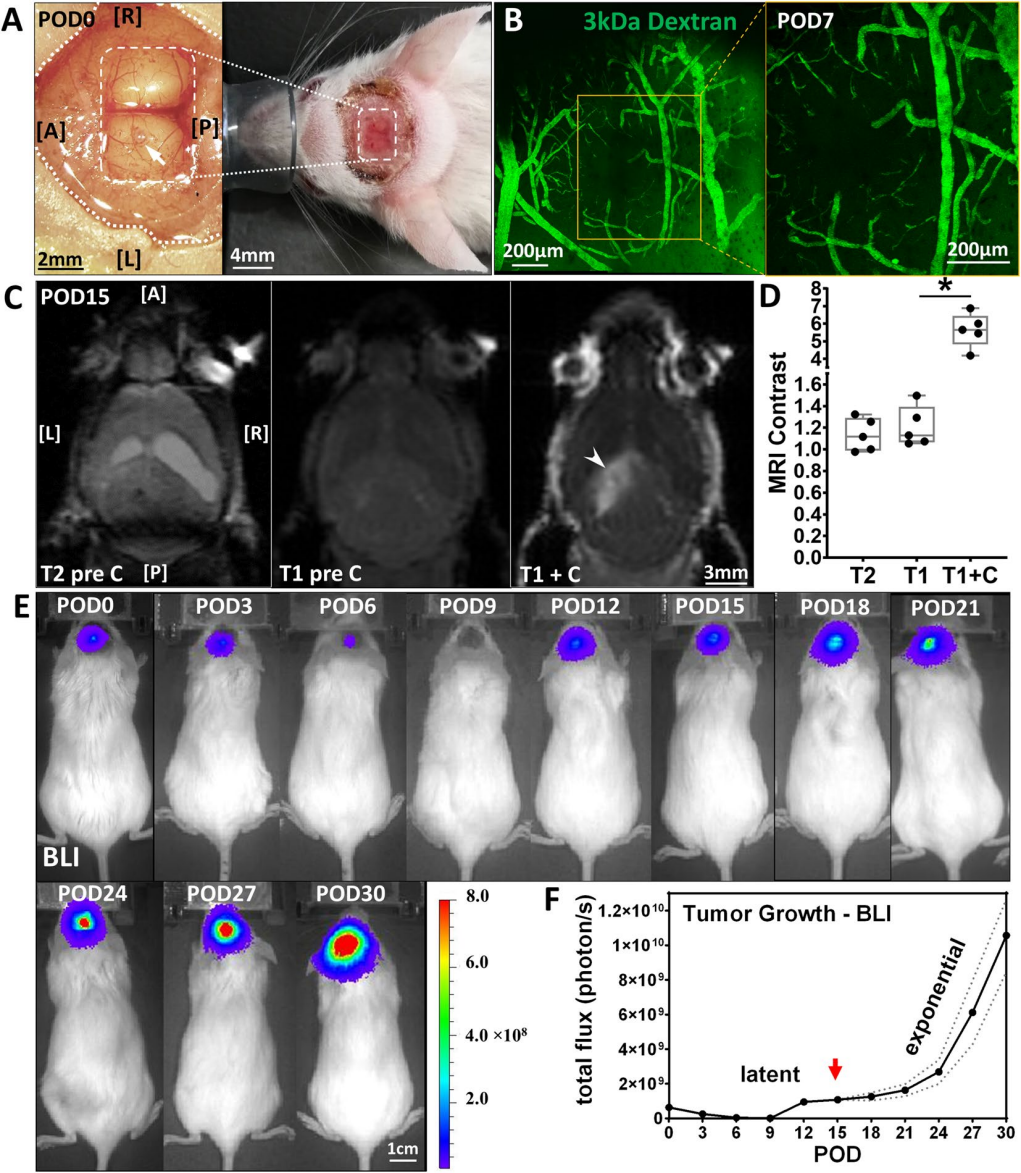
U251 orthotopic implant produces contrast enhancing HGG xenograft intracranial tumor in mice. (**A**) Photograph of U251 implanted (*arrow*) in mouse brain before (*Left*) and after (*Right*) cranial window (*dashed box*) placement in surgical field (*dotted lines*) on POD0. A: anterior; P: posterior; L: left; R: right. (**B**) Maximum intensity projection (MIP) image (2 × 2 stitched tiles) across 0-300 µm below cranial window 30 s after intravenous injection of 3 kDa fluorescein-dextran on POD7. *Yellow box*: a single-tile MIP image at implant site. (**C**) T2-weighted (pre-contrast) and T1-weighted (pre- and post-contrast) MRI scans from a mouse with U251 glioma xenograft on POD15. *Arrowhead*: non-enhancing region within tumor. (**D**) MRI contrast of tumor against normal (Mean ± SEM) revealed significant statistical difference (*P < 0.001) between T1-weighted images with and without contrast injection. (**E**) Bioluminescence imaging (BLI) of glioma xenograft growth in mice over 30 days. (**F**) Tumor size measured by bioluminescence grew exponentially after a latent period. *Dotted lines*: standard error of the mean. *Arrow*: beginning of exponential growth.

U251 cells implanted (*arrow*) in the left hemisphere of mouse brains (*n* = 5) were optically accessible through a glass cranial window (*white box*), Fig. 2A. The implant site was examined with intravital microscope on POD7 to confirm the xenograft was growing close to the brain surface (0–300 µm), Fig. 2B. Intravenously injected dextran (3 kDa) was confined to the lumen of the blood vessels as a vascular tracer and suggested an intact blood–brain barrier (BBB). The tumor area revealed marked hyperintense signal in T1-weighted MRI scans with gadolinium contrast on POD15, indicating a disrupted BBB to gadolinium (molecular weight: 1058 Da), and the non-enhancing region (*arrowhead*) within the tumor corresponding to the focus of necrosis, Fig. 2C. The average tumor volume reached 12.1 ± 1.9 mm^3^ with 3.5 ± 1.2 mm in the greatest dimension. Significant post contrast enhancement between tumor and normal brain was seen on T1 imaging, from 1.21 ± 0.19 to 5.64 ± 0.98 (*P* = 0.00057, n = 5), Fig. 2D. Minimal contrast (1.13 ± 0.15) was present in the tumor area on T2-weighted MRI, indicating the absence of hydrocephalus. Tumor growth was monitored for one month via bioluminescence, Fig. 2E. Exponential growth was followed for a latency period of 12 to 18 days, with 16.7 ± 1.38-fold change from POD0 to POD30, Fig. 2F. Subsequent imaging took place at the beginning of exponential growth on POD15 (*arrow*).

### Panitumumab-IRDye800 detects HGG in mice in vivo and ex vivo on macroscopic near-infrared fluorescence imaging

**Figure 3:**
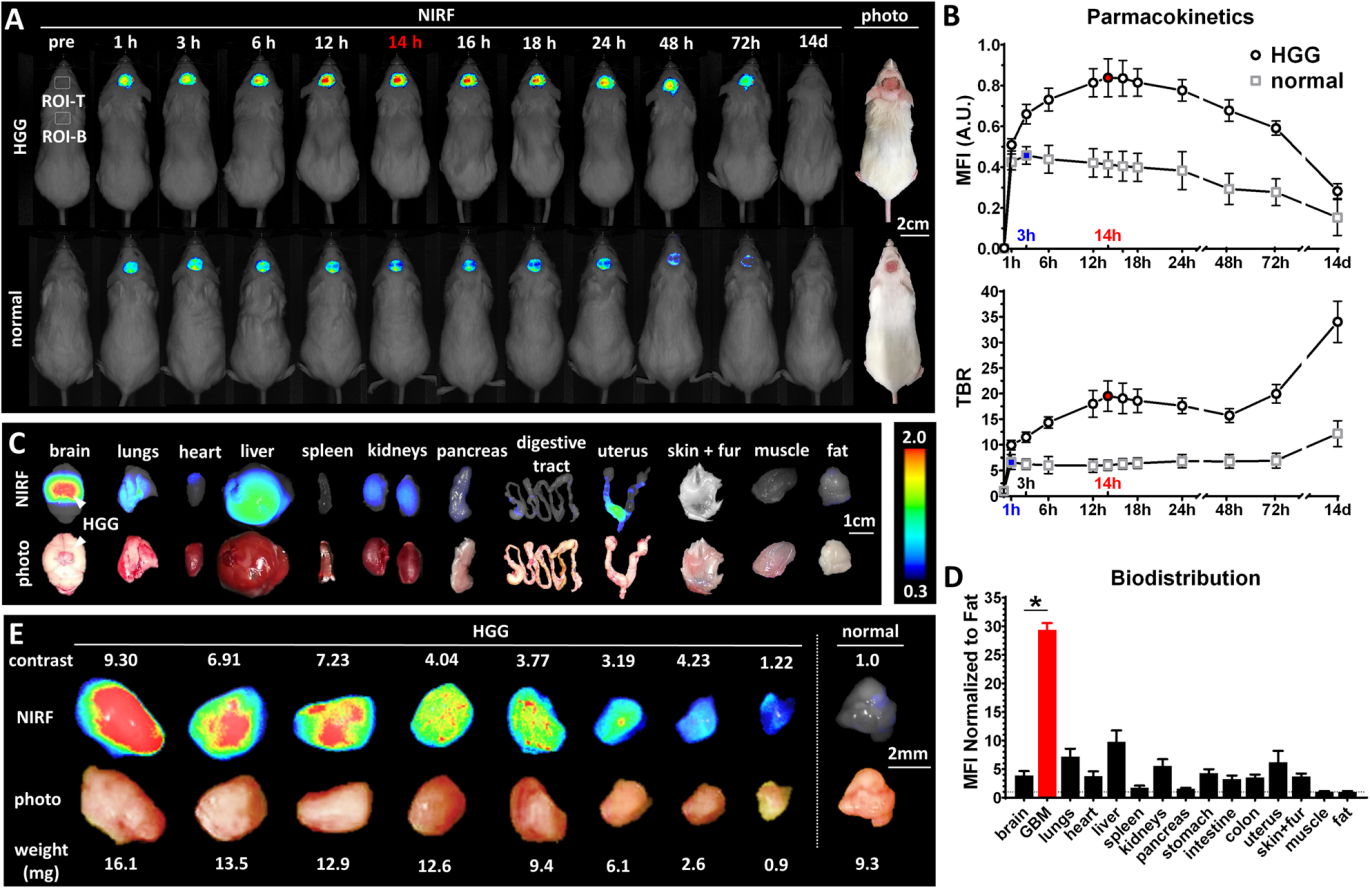
Pharmacokinetics and biodistribution of panitumumab-IRDye800 in vivo and ex vivo. (**A**) In vivo near-infrared fluorescence imaging (NIRF) of mice bearing orthotopic HGG xenografts or normal mice (sham injection of saline) with cranial windows before and after panitumumab-IRDye800 injection over the period of 14 days. *Dotted boxes*: regions of interest (ROI) for mean fluorescence intensity (MFI) and target-to-background ratio (TBR) measurements (T: tumor; B: background). (**B**) Pharmacokinetics of panitumumab-IRDye800 in normal and HGG bearing mice, with standard error of the mean. *Red* and *blue*: peak values and corresponding time points for HGG and normal mice, respectively. (**C**) NIRF images and corresponding photos of organs removed from HGG xenograft-bearing mice at 14 h post panitumumab-IRDye800 injection. *Arrows*: HGG xenograft. (**D**) Biodistribution in resected organs was quantified as MFIs (± SEM) normalized to that of fat, *P < 0.001 (*n* = 5) between normal brain and HGG. (**E**) NIRF images and corresponding photos of serial bisected HGG tumor pieces and normal brain resected from mouse brains 14 h post panitumumab-IRDye800 injection. TBR: MFI of each HGG tissue piece divided by MFI of normal brain (TBR of normal brain is set to 1.0 for each mouse).

In mice bearing contrast enhancing HGGs (*n* = 5), intratumoral fluorescence steadily increased after panitumumab-IRDye800 injection, peaking at 14 h, and was maintained at a high level from 12 to 18 h before tapering off, Fig. 3A. Mean fluorescence intensity (MFI) reduced to half of its 14-h peak after 9 days, while the target-to-background ratio (TBR) reached a high point of 19.5 ± 1.3 at 14 h and kept increasing after reaching a minimum at 48 h, indicating a faster clearance of panitumumab-IRDye800 from normal tissue than tumor, Fig. 3B. In comparison, much lower panitumumab-IRDye800 accumulated in the brain of normal mice, reaching a peak at 3 h (MFI = 54.6 ± 6.1% compared to that of HGG bearing mice, TBR = 6.6 ± 0.6) and returned to the baseline after two weeks, Fig. 3A,B. In resected organs of tumor bearing mice (*n* = 5), panitumumab-IRDye800 was distributed in liver, lungs, kidneys and uterus (MFI > 5 times normalized to fat), Fig. 3C,D. A high concentration of panitumumab-IRDye800 was found to be specifically accumulated in HGG xenografts compared to surrounding normal brains (29.4 ± 2.6 vs. 3.9 ± 1.8 normalized to fat, contrast against normal brain = 7.6 ± 1.5, *P* = 8.03 × 10^–8^), Fig. 3D. Resected HGG tumors were serially bisected and the contrast between tumor pieces against normal brain ranged between 1.22 and 9.30, Fig. 3E. Tumor pieces as small as 0.9 ± 0.3 mg could be readily detected on fluorescence imaging.

### Tumor biomarkers correlate with intratumoral and molecular specific uptake of panitumumab-IRDye800

**Figure 4:**
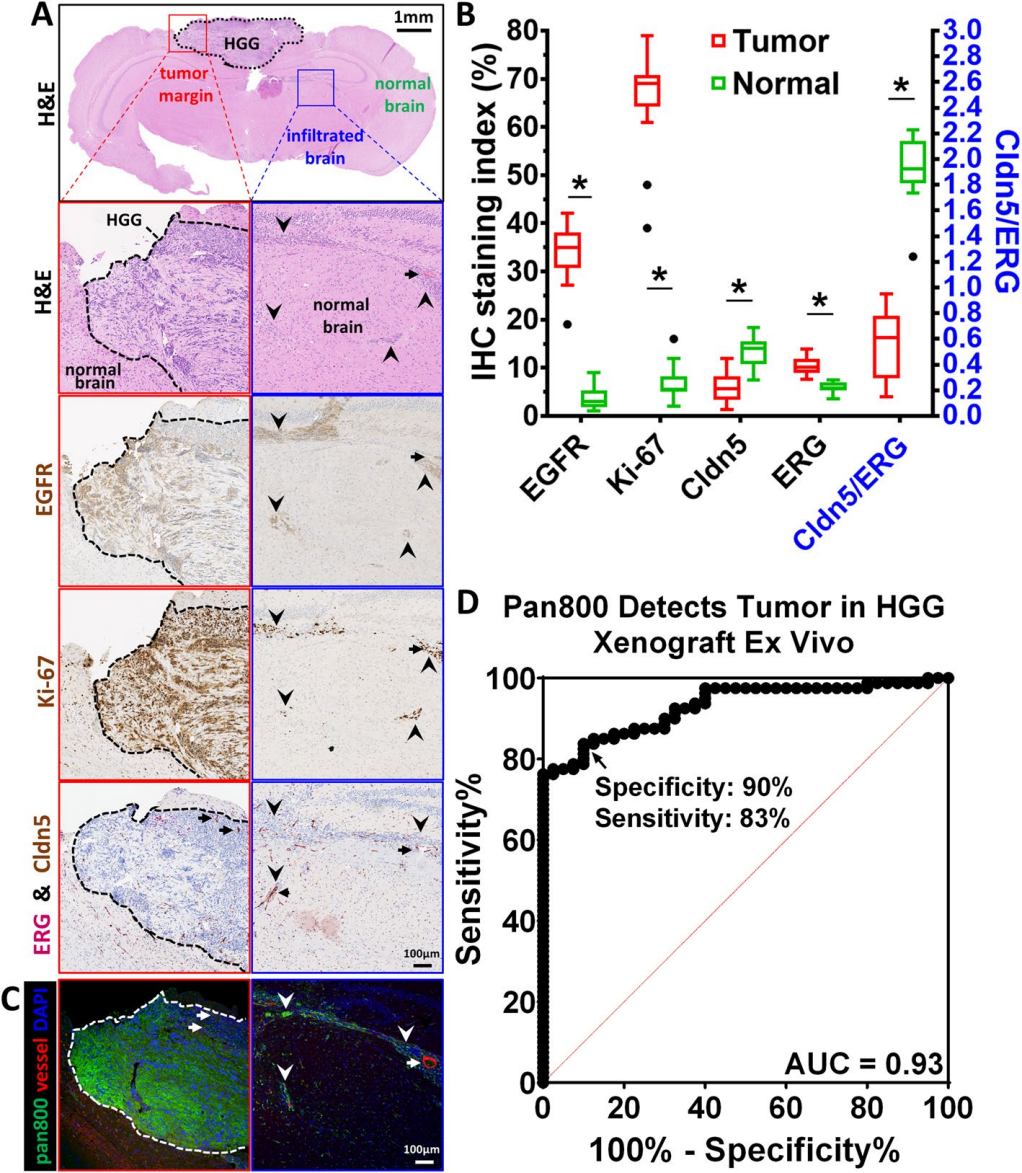
Tumor biomarkers and microscopic NIRF imaging of panitumumab-IRDye800 in HGG xenograft. (**A**) Histology (H&E) and immunohistochemistry staining of 4 µm sections of HGG xenograft bearing mouse brains 14 h post panitumumab-IRDye800 injection. EGFR: epidermal growth factor receptor; Ki-67: nuclear protein associated with cellular proliferation; Cldn5: tight junction protein, claudin-5; ERG: erythroblast-transformation-specific related gene (a vascular endothelial marker). *Red boxes*: primary HGG core and normal brain margin (*black dotted line*); *Blue boxes*: HGG cells infiltrating normal brain; *Arrows*: blood vessels; *arrowheads*: infiltrating HGG tumor cells. (**B**) IHC staining index (left Y axis) and Cldn5/ERG ratio (right Y axis, blue) of tumor areas (*n* = 16) and normal brains (*n* = 10), respectively (*P < 0.001). (**C**) NIRF microscopic imaging of intratumoral uptake of panitumumab-IRDye800 (pan800, green) in HGG mouse brain sections in the same field of view as (**A**). (**D**) Fluorescence intensity of panitumumab-IRDye800 fluorescence distinguishes tumor from normal brain in (**C**) with 90% specificity and 83% sensitivity. AUC: area under the curve; ROC: receiver operating characteristic.

In H&E stained mouse brain sections, tumor cells were identified by their high nucleus to cytoplasm ratio, nuclear and cytoplasmic abnormal morphology, and their pleomorphism, Fig. 4A. Images of immunohistochemical staining were segmented into positive and negative pixels according to staining intensity, Supplementary Fig. S2, and quantified as staining index. HGG xenograft and infiltrating tumor cells expressed a modest level of EGFR (staining index: 34.2 ± 1.9%, *P* = 7.49 × 10^–15^), Supplementary Fig. S2, and were highly proliferative (Ki-67 staining index: 66.3 ± 2.5%, *P* = 1.28 × 10^–15^) compared to surrounding normal brain (staining index: 3.6 ± 0.8% and 7.1 ± 1.3% and for EGFR and Ki-67, respectively), Fig. 4A,B. Tight-junction protein at the blood–brain barrier, claudin-5 (Cldn5, brown, cytoplasmic), was reduced in the tumor core (*P* = 4.65 × 10^–6^), Supplementary Fig. S2, and elevated endothelial cell marker, erythroblast-transformation-specific related gene (ERG, magenta, nuclear), detected hypervascularity within infiltrating tumor beds (*P* = 2.73 × 10^–7^). A diminished (29.4% of normal brain, *P* = 6.67 × 10^–12^) tight-junction protein per unit of vasculature (Cldn5/ERG) was found to be consistent with the compromised integrity of the BBB on contrast enhancing MRI. Intratumoral delivery of panitumumab-IRDye800 in near-infrared fluorescence microscopic images in areas of open BBB allowed antibody-sized molecules to cross. Furthermore, a correlation (Pearson correlation coefficient r = 0.88, *P* = 2.76 × 10^–9^) between fluorescence intensity, Fig. 4C, and EGFR immunoreactivity, Fig. 4A, validated molecular specific binding of panitumumab-IRDye800. Minimal panitumumab-IRDye800 delivery was detected in normal brain characterized by a normal BBB and resulted in high contrast (TBR = 9.1 ± 1.7). NIR fluorescence from panitumumab-IRDye800 was able to detect tumor at 90% specificity and 83% sensitivity (area under the curve = 0.93), Fig. 4D.

### EGFR protein is commonly expressed in a variety of human high-grade brain tumors

**Figure 5:**
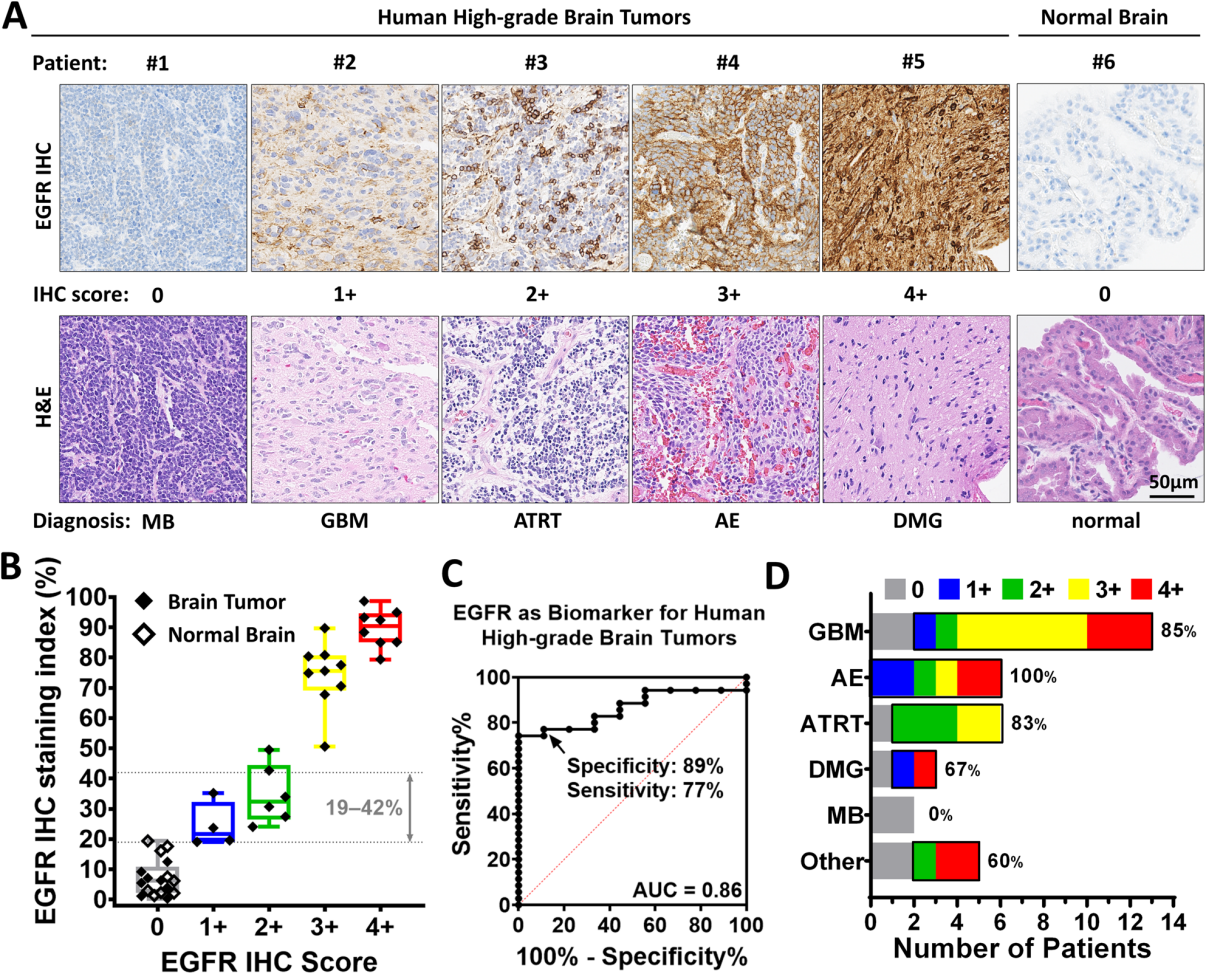
EGFR expression across human high-grade brain tumors. (**A**) Representative microscopic images of EGFR IHC staining scored as 0, 1 +, 2 +, 3 + and 4 + by neuropathologists on human high-grade brain tumor and normal brain tissue sections. H&E: corresponding histology staining of each tissue. MB: medulloblastoma; GBM: glioblastoma multiforme; ATRT: atypical teratoid rhabdoid tumor; AE: anaplastic ependymoma; DMG: diffuse midline glioma. (**B**) EGFR IHC staining index quantified from HGG patient tissues in (**A**). (**C**) EGFR distinguished human HGG tumor from normal brain as a biomarker in (**B**) with 89% specificity and 77% sensitivity. AUC: area under the curve; ROC: receiver operating characteristic. (**D**) Percentage of patients with EGFR positive expression (scored 1 + to 4 +) in high-grade brain tumor disease subtypes. GBM (*n* = 13), AE (*n* = 6), ATRT (*n* = 6), DMG (*n* = 3), MB (*n* = 2) and other: anaplastic astrocytoma (4 +, *n* = 1), pleomorphic glial neoplasm (4 +, *n* = 1), anaplastic oligodendroglioma (2 +, *n* = 1), ganglioneuroblastoma (0, *n* = 1) and anaplastic ganglioglioma (0, *n* = 1).

To assess the potential of detecting EGFR with panitumumab-IRDye800 on imaging in HGG patients, EGFR protein expression was immunohistochemically characterized in human tissues. 59 tissue samples from a total of 35 (2.9%) patients were included in the study, Supplementary Table S1, with an even split between the genders (M/F = 17/18). EGFR protein immunochemistry (IHC) reactivity was heterogeneous and was scored (0, 1 +, 2 +, 3 + and 4 +) by two board-certified neuropathologists, Fig. 5A. 27 (77.1%) of the 35 patients expressed EGFR protein at levels either equivalent (1 + & 2 + staining index: 18–49%) or above (3 + & 4 + staining index: 50–97%) that in mouse HGG xenografts (staining index: 19–42%), Fig. 5B. Tissues from nine patients also contained normal brain structures (e.g. choroid plexus and cerebellum) with little, if any, non-specific EGFR immunoreactivity. As a biomarker, EGFR protein expression distinguished tumors against normal brain with high specificity (89%) and sensitivity (77%) at 18.3% cutoff IHC staining index (likelihood ratio = 6.9), Fig. 5C. A majority of patients in various high-grade brain tumor disease subgroups expressed positive EGFR (anaplastic ependymoma, 100%; glioblastoma, 85%; atypical teratoid rhabdoid tumor, 83%; and diffuse midline glioma, 67%), Fig. 5D. A substantial number of these patients (anaplastic ependymoma and ATRT, 50%; diffuse midline glioma, 33%; and glioblastoma, 15%) expressed EGFR at a modest level (IHC score: 1 + and 2 +) comparable to that found in the mouse xenografts. Two cases of medulloblastoma in this study expressed no EGFR. 60% of less common diagnoses (grouped as “Other”) expressed positive EGFR. These results suggest that a large number of HGG patients are expressing EGFR equal to or above the level detectable by panitumumab-IRDye800 imaging and could potentially benefit from fluorescence image-guided surgery.

### Clinical features of EGFR positive HGG patients

Since patients with positive EGFR protein expression are more likely to benefit from EGFR-targeted fluorescence imaging, the focus herein was to identify patient characteristics or tumor features associated with positive EGFR protein expression. Of the 35 patients, 3 (15.8% of the 19 with molecular diagnosis) had *EGFR* gene amplification while 27 (77.1%) presented positive EGFR protein expression, Fig. 5C. These tumors were also stratified by *IDH* and tumor protein 53 (*TP53*) mutations to determine the influence of these mutations on the molecular profile of a given tumor. Clinical characteristics of patients with and without EGFR protein expression, are shown in Table 1. Patients with positive EGFR protein expression were older at diagnosis (median 24 versus 5 y, *P* = 0.0059), had tumors located more frequently in the cerebral hemisphere (74% vs. 37.5%, *P* = 0.019) and were more likely to be *IDH* wildtype (94% vs. 0%, *P* = 0.0048). Over two thirds (69.2%) of pediatric patients (age < 18 years) had HGGs that expressed detectable EGFR protein on IHC. Differences in gender, tumor size, classification, WHO grade, aggressiveness (Ki-67 proliferation index) and recurrence history were not observed between the two groups; the incidence of *TP53* mutation and *EGFR* gene amplification were also similar.

**Table 1:** Patient demographics.

	EGFR −ve (*n* = 8)	EGFR + ve (*n* = 27)	*P* value
**Age, years**			*0.0059^a^
Mean (SD)	5 (5)	24 (23)	
Median	5	15	
Q1, Q3	1, 9	6, 44	
Range	0–12	1–68	
**Sex**			0.13^b^
F	6 (75%)	12 (44%)	
M	2 (25%)	15 (56%)	
**Tumor size, cm**			0.093^a^
Mean (SD)	6.6 (2.8)	4.6 (1.8)	
Median	5.7	5	
Q1, Q3	4.3, 8.5	3.6, 5.4	
Range	3.8–11.2	1.0–8.7	
**Location**			*0.019^b^
Cerebral hemisphere	3 (37.5%)	20 (74%)	
Posterior fossa	4 (50%)	2 (7%)	
Thalamus, ventricle & spinal cord	1 (12.5%)	5 (19%)	
**Histological grading**			0.33^b^
WHO Grade III	1 (12.5%)	8 (30%)	
WHO Grade IV	7 (87.5%)	19 (70%)	
**Tumor classification**			0.20^b^
Diffuse astrocytic and oligodendroglial^c^	3 (37.5%)	15 (56%)	
Embryonal^d^	4 (50%)	5 (19%)	
Ependymal, neuronal and others^e^	1 (12.5%)	7 (26%)	
**Tumor medical history**			0.68^b^
De novo	6 (75%)	22 (81%)	
Recurrent	2 (25%)	5 (19%)	
**Ki-67 proliferation index, %**			0.71^a^
Mean (SD)	22 (21)	22 (16)	
Median	15	18	
Q1, Q3	5, 38	11, 33	
Range	1–54	2–59	
**Molecular features**			
*IDH* mutation	1 (100%)	1 (6%)	*0.0048^b^
*IDH* wildtype	0	15 (94%)	
*IDH* unknown	7	11	
*TB53* mutation	3 (50%)	9 (50%)	1.00^b^
*TP53* wildtype	3 (50%)	9 (50%)	
*TP53* unknown	2	9	
*EGFR* amplification	0	3 (21%)	0.26^b^
*EGFR* unamplied	5 (100%)	11 (79%)	
*EGFR* unknown	3	13	

^a^Wilcoxon rank-sum test.

^b^Pearson’s chi-squared test.

^c^Glioblastoma multiforme (GBM), diffuse midline glioma (DMG), anaplastic astrocytoma, anaplastic oligodendroglioma.

^d^Medulloblastoma (MB), ganglioneuroblastoma, atypical teratoid rhabdoid tumor (ATRT).

^e^Anaplastic ependymoma (AE), anaplastic ganglioglioma, pleomorphic glial neoplasm.

## Discussion

Intratumoral and intertumoral EGFR expressions in HGGs are known to be heterogeneous^14^. Many preclinical imaging studies were conducted on subcutaneous implants with monolithically highly positive expression of the molecular target of interest or a small number of specific patient-derived xenografts without relating their target expression level with the larger patient population. In this preclinical study, U251 cells were specifically chosen, from in vitro immunofluorescence assays, to establish the orthotopic brain tumor model in order to assess the imaging performance of panitumumab-IRDye800 in a challenging yet realistic environment where EGFR expression in tumors is more modest. As 77.1% of all human HGG tissues tested expressed EGFR at equal or higher levels, it is reasonable to believe that panitumumab-IRDye800 would be able to detect them in vivo as well given the right conditions.

The advantage of panitumumab-IRDye800 is that this agent, due to its targeting specificity, could selectively bind to a tumor-specific biomarker of HGGs. When tested in the same animal model, panitumumab-IRDye800 demonstrated over 10% higher specificity for tumor and 30% higher comprehensive TBR than those of 5-ALA^15^. As an imaging probe for fluorescence-guided surgery, panitumumab-IRDye800 could potentially outperform 5-ALA which suffers from limited imaging contrast and low negative predicting value (16.7% for HGG)^16^. Diffuse pink fluorescence from PPIX has been detected beyond the contrast-enhancing tumor components in glioblastoma which contained either infiltrating tumor cells of medium density or edematous brain tissue^17^. PPIX fluorescence in fluid-filled, nonmalignant tissue may be one source of false positives found with 5-ALA^18^. On the other hand, EGFR-amplified cells are preferentially located at the infiltrating edge rather than distributed uniformly within GBM tumors^19^, thus more likely to provide the higher contrast and tumor specificity in order to delineate the tumor tissue at the infiltrative margin. Distribution of panitumumab-IRDye800 in resected tumor tissues could also have potential therapeutic implications such as monoclonal antibody delivery for targeted therapy although efficacy studies are beyond the scope of this study.

The fluorescence signal in mouse brain tumors reached its peak at 14 h after systemic panitumumab-IRDye800 injection. The time frame of 12 to 18 h after panitumumab-IRDye800 infusion at this particular dose is promising for clinical translation of fluorescence guided surgery, since patients can be infused with the imaging agent on the day before surgery^10,20^. This is a favorable window for imaging and gives enough time for observation of potential adverse effects. In comparison, FDA (U.S.) approved intraoperative optical imaging agent for glioma surgery, 5-ALA^21^, is administered orally 2 to 4 h before anesthesia, which can be logistically challenging to align clinical workflow with the more narrow optimal imaging window. Similarly for passive fluorescent probes, fluorescein sodium has a narrow imaging window of 1–5 h (peaking at 2 h) post infusion while the observed duration of indocyanine green (ICG) fluorescence in brain tumor surgery studies was limited, with a peak at about 10 min^22,23^. The prolonged high TBR of panitumumab-IRDye800 measured over two days can better accommodate changes in clinical workflow, which is another desirable feature in addition to its targeting specificity. In vivo imaging would be less than ideal after 48 h, since by then, overall fluorescence intensity will have dropped significantly despite TBR continuing to increase due to faster clearing of non-specific panitumumab-IRDye800 binding.

The EGFR specific distribution of panitumumab-IRDye800 was associated with increased vasculature density and reduced BBB tight junction protein expression in contrast enhancing HGG xenografts in mice. This result was consistent with a previous clinical study where intratumoral uptake of cetuximab-IRDye800CW was observed in contrast-enhancing HGGs in two patients, but not in a third patient with an astrocytoma that on MRI also did not demonstrate any contrast enhancement^24^. Intraoperative dyes such as fluorescein sodium and ICG have smaller molecular sizes and can be delivered across disrupted BBB. Although fluorescein-guided glioma resection has emerged due to its safety profile and low cost ($3181 incremental cost per quality-adjusted life-year) since 1948^22,25^, fluorescein is not tumor cell-specific and fluorescein use in brain tumor surgery is not currently an FDA-approved use^26–29^. Some researchers warned that fluorescein application outside of clinical studies is premature and emphasized possible false positive and false negative staining during surgery^26,30^. Taken together, these results suggest that compromised BBB integrity, indicated by contrast enhancing tumor on MRI with small molecule gadolinium, may predict intratumoral delivery of antibody-sized molecules, although further work is needed to validate this finding and elucidate the specific mechanism that may be involved in this phenomenon.

In addition to its affinity for HGGs, the optical properties of fluorescence probes also contribute to the accuracy of fluorescence-guided resection. Violet blue light, which excites 5-ALA-induced protoporphyrin IX (PpIX), is also absorbed by hemoglobin. As a result, oozing blood in the tumor cavity and overlying soft tissues can disturbs excitation of 5-ALA-induced PpIX in residual tumor and decrease visible fluorescence^23^. A large percentage of tumor-positive biopsy sites (∼40%) that were not visibly fluorescent under the operating microscope had levels of PpIX concentration greater than 0.1 µg/mL, indicating that significant PpIX accumulation exists below the detection threshold of current fluorescence imaging^31^. Photo bleaching is another recognized issue of PpIX fluorescence, which plays a minor role during 5-ALA fluorescence-guided surgery since new tissue layers are exposed during tumor resection^18^. On the contrary, light in the near-infrared window (650–1350 nm) has maximum depth of penetration in tissue. Panitumumab-IRDye800 concentration as low as 13 pM was detectable in intraoperative imaging of head-and-neck cancer^32^, almost 60 times more detectable than PpIX. Moreover, phthalocyanine-based fluorescence dyes such as IRDye800 have high quantum-yield and photostability to allow even single molecule imaging^33^.

Population subgroups depending on intrinsic factors such as age, gender and ethnicity are commonly and intentionally included in efficacy and safety evaluation of clinical trial outcomes^34^. However, patients in HGG disease subgroups with lower incidence rates but at equally high-risk, for whom effective therapeutic concepts are desperately needed, tend to be underrepresented in clinical brain imaging studies^24,35,36^. As the most frequent malignant brain tumor in adults, glioblastoma accounts for 45–50% of all primary malignant brain tumors and occurs 30 times as frequently as anaplastic oligodendroglioma^37^. On the other hand, ATRT and diffuse midline glioma are rarely seen in adults but account for 10% and 20% of all pediatric primary tumors, respectively^38,39^. The variable clinical outcome of anaplastic ependymomas in both adults and children primarily depends on the extent of surgical resection and molecular group, pointing to the clinical relevance of molecular classification^40^. Ongoing clinical trials for fluorescence guided surgery of brain tumors using EGFR antibody or affibody (NCT03510208, NCT02901925 and NCT04085887) indicate the active exploration of molecular targeting strategy for detecting gliomas in both adults and children. This preclinical study examined the dynamic tumor fluorescence change over time with consistent imaging window and tumor size across multiple subjects, which can be impractical to conduct in human studies. Our preliminary findings on the frequent presence (> 50%) of EGFR protein expression across multiple HGG disease subgroups are encouraging for including patients with broader age groups and diverse HGG subtypes (such as anaplastic ependymoma, ATRT and diffuse midline glioma, which predominate in children) in future clinical trials for imaging and antibody treatment therapy.

Achieving satisfactory intraoperative imaging performance using open-field optical imaging instruments has been challenging despite the presence of adequate in vivo and ex vivo imaging contrast in preclinical studies where closed-field fluorescence devices were used for imaging probe development^41^. FDA-approved optical imaging devices vary in dynamic range and detection sensitivity, and are subject to a complex lighting environment of the operating room and clinical workflow constraints. The Pearl Trilogy imaging platform for this preclinical study has been used in various clinical trials for fluorescence-guide surgery to evaluate fluorescence signal of ex vivo patient tissues in the operating room^20,42,43^. Thus the ex vivo tissue detection sensitivity (0.9 mg) in the mouse model can be a realistic indication of imaging performance in the operating room under similar conditions. The high contrast (TBR = 7.6 ± 1.5) measured in resected tumor tissues is also encouraging for the clinical application of an intraoperative pathological assessment of tumor margins. Testing tumor imaging agents in preclinical studies under lighting conditions which mimic the operative microscope or ambient lighting and with other imaging instruments would be very valuable for further evaluation of their translational potential.

Although molecular targeting probes are attractive and technologically advanced, their benefit and cost effectiveness compared to already existing 5-ALA and fluorescein for fluorescence-guided resection are yet to be proven in clinical studies. Combining the probes with molecules for secondary goals such as chemotherapy, photosensitization, and others may be advantageous. The results on EGFR expression in human HGGs are based on limited number of tissue samples and may be affected by the fact that rare HGG subgroups were referred to Stanford more often than their natural incidence in the population. The application of antibody-based imaging strategy on pediatric and rare HGG disease subgroups that express EGFR still need to be validated in animal tumor models and clinical studies.

In conclusion, significant imaging contrast was observed 14 h after systemic panitumumab-IRDye800 administration in HGG xenografts with modest EGFR protein expression. Both target and tumor specific probe uptake was confirmed microscopically. In the majority of patient HGG tissues, EGFR protein expression levels were equivalent to or higher compared to that in the preclinical model, suggesting their detectability with intraoperative EGFR targeted imaging by panitumumab-IRDye800. Older patients with IDH-wildtype cerebral hemisphere tumors tend to express greater levels of EGFR protein. These findings demonstrate the feasibility to employ an EGFR-targeting antibody for fluorescence-guided surgery of HGGs, as well as the utility of clinical profiling in stratification of HGG patients most likely to benefit from antibody-based imaging strategies.

## Methods

### Patients

This single-institution study was approved by the Stanford University Institutional Review Board (protocol #12625), and informed consent was obtained from all participants and/or their legal guardians. All experiments were performed in accordance with relevant guidelines and regulations. Central pathology review by the Stanford University Department of Pathology followed the 2016 WHO criteria. A total of 1197 patients were identified in the Stanford University archives with a primary diagnosis of high-grade brain tumors (WHO grade III or IV) between 2006 and 2019. 1197 randomized integers were generated (https://www.random.org/integers/) with values between 1 and 100 (both inclusive) and assigned to de-identified eligible patients. 5% of eligible patients (n = 60) were included in the study (those with numbers 1–5). Cases with either scant tissue or incomplete medical records were excluded, resulting in the 35 patients in the study. Two board-certified pathologists reviewed hematoxylin and eosin (H&E) slides to outline regions of viable tumor and normal brain. Peripheral cases and those with scant tissues (size < 1 mm) were excluded.

### Cells

Human glioma-derived tumorigenic cell lines, U251 (luc +, from Dr. Lawrence Recht, Stanford University), H37, D2159 and D270 (from Dr. Vidya Chandramohan, Duke University) were maintained in improved MEM zinc option medium (Gibco) as previously described^44^. Head-and-neck squamous cell carcinoma (SCC-1, University of Michigan) and human embryonic kidney cells (293 T, ATCC) were positive and negative controls for EGFR expression, respectively. All cultures were routinely subjected to mycoplasma testing and only used for experiments when confirmed negative. Routine short tandem repeat analysis was performed using the Geneprint 10 system (Promega) to ensure cell line identity. Growth and morphology were observed with bright field (Evos XL Core, Life Technologies) and phase contrast (BZ-X710, Keyence).

### Immunofluorescence analysis

Cells were fixed on glass slides in 4% paraformaldehyde for 15 min followed by one hour blocking (2% horse serum and 5% goat serum diluted in 1% BSA). They were incubated overnight at 4 °C with either a commercial unlabeled EGFR antibody (1:2.5, RM-2111-RQ, rabbit-anti-human, Thermo Scientific) followed by secondary antibody (Alexa Fluor 594, Invitrogen) in blocking buffer for one hour at room temperature, or a NIR-labeled human EGFR antibody, panitumumab-IRDye800 (5 nM, Leidos Biomedical Research Center, Frederick, MD). Slides were counterstaining with 4′, 6-diamidino-2-phenylindole (DAPI, 300 nM, Invitrogen), and imaged using a Zeiss LSM710 confocal fluorescence microscope or a custom-built fluorescence microscope (Leica)^45^. Mean fluorescence intensities (MFIs) from 10 cells per image in five images were normalized to that of the positive control (SCC-1) using ImageJ (v1.52p).

### Binding competition

U251 cells were incubated with 5 nM panitumumab-IRDye800 and unlabeled panitumumab in serial dilutions (0.005 nM–5 μM) overnight at 4 °C, in triple replicates. Fluorescent microscopic images were taken in five random field of views and MFIs from 10 cells per image was quantified with ImageJ (1.52p). Competitive binding curve was fitted to a logistic function equation for single-site binding Y = Bottom + (Top–Bottom)/(1 + 10^X-LogIC50^) where logIC50 = log(10^logKi^ × (1 + [Radioligand]/[HotKd])), Radioligand = 5 nM, HotKd = 0.12 nM^46,47^. Equilibrium dissociation constant Ki was determined as the half maximal inhibitory concentration (IC50) of unlabeled panitumumab that produces binding half-way between the upper and lower plateaus.

### Cranial window installation and orthotopic brain tumor mouse model

Animal experiments were carried out in accordance with Stanford University Institutional Animal Care and Use Committee guidelines (protocol #26548), and in compliance with the ARRIVE guidelines (PLoS Bio 8(6), e1000412, 2010). Cranial window installation and orthotopic brain tumor mouse model were established in twenty female NSG mice (005,557, Jackson Laboratory) as previously described^48^. U251 human xenografts were implanted (5 μL, 1 × 10^6^ cells/mL) and optically accessible through a 6.0 mm × 4.0 mm × 0.4 mm glass cranial window. Normal mice were prepared using the same protocol as controls, with sham injection of 5 μL saline instead of tumor cells. Tumor growth was monitored via bioluminescence (IVIS Spectrum, PerkinElmer) every three days for one month. Total photon flux (photons/s) over the mouse brains was quantified in Living Image (v4.5.5, PerkinElmer). To ensure growth of tumor close to the surface of the brain for subsequent imaging experiments, on postoperative day 7 (POD7), vascular elements in brain tumors were imaged through the cranial window with a two-photon intravital microscope (Nikon) following 3 kDa fluorescein-dextran injection (10 mg/mL, i.v., ThermoFisher). Excitation laser (920 nm) powers was modulated between 10 and 30 mW with depth (0–300 μm, step size = 5 μm). Images were reconstructed and analyzed with ImageJ (v1.52p).

### Magnetic resonance imaging

Anesthesia was induced with 5% isoflurane and maintained with 2% isoflurane in room air and oxygen mixed at 3:1 ratio. Five HGG bearing mice were imaged in a 7 T small-bore MR scanner (Bruker) with a Millipede RF coil (ExtendMR LLC) tuned to 298.06 MHz on POD15. T2-weighted images [repetition interval (TR) = 5 s, echo time (TE) = 33 ms, and echo train length = 8] were acquired without contrast injection. Areas of hyperintense signal in the T2-weighted images correspond to hydrocephalus due to blockage of the brain ventricles^49,50^. T2 also shows edema better which can also affect the microenvironment and interstitial pressure. Mice with areas of hydrocephalus were excluded from the study to reduce variability in tumor microenvironment and drug delivery. T1-weighted images [TR = 278 ms, TE = 2 ms] were taken before and after gadobenate dimeglumine (0.5 mmol/kg, i.v., molecular weight: 1058 Da, MultiHance). All images had 4 cm × 4 cm field of view, 0.5 mm slice thickness and 156 × 156 µm in-plane resolution.

### Near-infrared fluorescence imaging

Tumor bearing mice (*n* = 5) and normal mice (*n* = 5) with cranial windows were imaged in intervals over a period of 14 days (starting on POD15) after receiving 20 mg/kg panitumumab-IRDye800 (i.v.). Pharmacokinetics were measured as mean fluorescence intensity (MFI) and target-to-background ratio (TBR) calculated as MFI in each tumor region of interest, ROI-T, divided by MFI of normal tissue, ROI-B. Another five tumor bearing mice were sacrificed at the peak antibody-dye conjugate delivery time and MFIs of harvested organs were normalized to fat. Resected brain tumors were serial bisected into different sizes, weighed and imaged with normal brains. All images were collected in a near-infrared florescence imager (Ex/Em: 785/820 nm, Pearl Trilogy Imaging System, LI-COR) and processed with Image Studio (v5.2, LI-COR). Mouse brains (*n* = 5) removed 14 h after panitumumab-IRDye800 injection were formalin-fixed overnight and paraffin-embedded. Brain sections were rehydrated, incubated in DyLight 488 labeled tomato lectin (10 µg/mL, Vector Laboratories) for 30 min and counterstained with DAPI (300 nM, Invitrogen) before they were imaged with a custom-built fluorescence microscope (Leica)^45^. MFIs were measured from 10 randomly selected cells in three views.

### Immunohistochemical analysis

Formalin-fixed paraffin-embedded brain sections (4 μm thick) were incubated with primary antibodies after heat mediated antigen retrieval. All immunohistochemical procedures were performed following preprogramed standard protocol on an automatized histostainer (Dako Autostainer, Agilent) with positive and negative controls in each operation. Immunoreactivity was visualization with diaminobenzidine and magenta chromogens (Dako EnVision). A staining index was calculated as the product of intensity and fraction of positive tumor cells using Aperio ImageScope (Leica)^51^. The immunoreactivity of human brain tissues were scored by two board-certified pathologists as previously described^52^. Primary antibodies were: EGFR (prediluted, Thermo Scientific, RM-2111-RQ), Ki-67 (prediluted, Dako, GA62661-2), Claudin-5 (1:500, Thermo Scientific, 34–1600) and ERG (1:1000, Abcam, EPR3864). Secondary antibody: Envision FLEX + rabbit (linker) (prediluted, Dako, SM805) for EGFR; no secondary antibody was used for all other antibodies.

### Statistical analysis

Data are expressed as mean ± standard error of the mean (SEM). Paired and unpaired t-tests (two-tailed) were performed for T1-weighted MR imaging contrast and other group comparisons, respectively. Patient characteristics were compared between EGFR positive and EGFR negative groups using a Wilcoxon rank-sum test and Pearson’s chi-square tests (GraphPad Prism 8.0) as appropriate. Significance was defined at P < 0.05.

## Supplementary Information

Supplementary Information.
