# Supplementary material for: Molecular imaging of a fluorescent antibody against epidermal growth factor receptor detects high-grade glioma
Source: Sci Rep. 2021 Mar 11;11:5710. doi: 10.1038/s41598-021-84831-4 (PMC7952570; doi:10.1038/s41598-021-84831-4)

Supplementary information

Title

Molecular imaging of a fluorescent antibody against epidermal growth factor receptor detects high-grade glioma

Authors

Quan Zhou, PhD[1, 2], Johana C. M. Vega Leonel, PhD[1], Michelle R. Santoso, BS[3], Christy Wilson, PhD[1], Nynke S. van den Berg, PhD[2], Carmel T. Chan, PhD[4], Muna Aryal, PhD[4], Hannes Vogel, MD[5], Romain Cayrol, MD, PhD[5], Michael J. Mandella, PhD[4, 6], Frank Schonig, BS[6], Guolan Lu, PhD[2], Sanjiv S. Gambhir, MD, PhD [4], Michael E. Moseley, PhD [4], Eben L. Rosenthal, MD [2, 7], Gerald A. Grant, MD [1*]

Supplementary Table S1. Demographic and diagnostic information on high-grade glioma (HGG) cases

patient#	Sex	Age (year)[a]	Size (cm)[b]	Location	Diagnosis	WHO Grading	EGFR IHC Grade[c]	EGFR gene amplification[d]	P53 abnormality[d]	IDH1 mutation[d]
1	F	9W	9.5	left hemisphere	glioblastoma multiforme	IV	-ve	—	—	X
2	F	9M	6.2	brain stem	glioblastoma multiforme	IV	-ve (n=2)[e]	O	X	—
3	F	12 (D)	3.5	right frontal lobe	glioblastoma multiforme	IV	2.5+	O	X	O
4	M	15	5.4	right frontal lobe	glioblastoma multiforme	IV	1+	O	X	O
5	M	15 (D)	5.2	right frontal lobe	glioblastoma multiforme	IV	3.5+	X	—	O
6	M	17 (D)	4.2	left temporal lobe	glioblastoma multiforme	IV	2+	—	X	O
7	F	41	5.1	right frontal lobe	glioblastoma multiforme	IV	3+	—	O	O
8	M	45	1.5	right temporal lobe	glioblastoma multiforme	IV	3+ (n=3)	—	X	O
9	M	55 (D)	5.3	left temporal lobe	glioblastoma multiforme	IV	3+ (n=3)	X	X	O
10	F	60	4.4	right frontal lobe	glioblastoma multiforme	IV	3+ (n=3)	—	—	O
11	F	67	3.6	left parietal lobe	glioblastoma multiforme	IV	3+ (n=3)	—	—	O
12	F	67	5.8	left parietal lobe	glioblastoma multiforme	IV	4+ (n=3)	X[f]	O	O
13	M	68	5.0	right frontal lobe	glioblastoma multiforme	IV	4+ (n=3)	—	—	—
14	M	4	1.0	left occipital lobe	anaplastic ependymoma	III	3+	O	O	—
15	F	5 (D)	4.0	right frontal lobe	anaplastic ependymoma	III	4+ (n=3)	O	O	—
16	F	6 (D)	1.6	right frontal lobe	anaplastic ependymoma	III	2+	—	—	—
17	F	10	4.5	left frontal lobe	anaplastic ependymoma	III	1+	O	O	—
18	M	15	5.0	posterior fossa	anaplastic ependymoma	III	1+	O	O	O
19	M	17	5.8	right temporoparietal	anaplastic ependymoma	III	4+	—	O	O

20	M	7M	4.0	posterior fossa	atypical teratoid rhabdoid tumor	IV	-ve (n=2)	O	X	—
21	F	10M (D)	8.0	right parietal lobe	atypical teratoid rhabdoid tumor	IV	2+	—	X	—
22	M	1 (D)	6.4	posterior fossa	atypical teratoid rhabdoid tumor	IV	2+	—	—	—
23	M	1	5.1	right frontal lobe	atypical teratoid rhabdoid tumor	IV	3+	—	—	—
24	M	4 (D)	8.7	left lateral ventricle	atypical teratoid rhabdoid tumor	IV	2+	—	—	—
25	F	5 (D)	3.5	cerebellum	atypical teratoid rhabdoid tumor	IV	3+	—	—	—
26	F	5	5.2	posterior fossa	medulloblastoma	IV	-ve (n=3)	O	O	—
27	M	11	4.4	posterior fossa	medulloblastoma	IV	-ve (n=3)	—	O	—
28	M	10 (D)	3.4	right thalamus	diffuse midline glioma	IV	4+	O	X	—
29	F	12	3.8	left thalamus	diffuse midline glioma	IV	-ve	O	X	—
30	F	14 (D)	6.0	spine	diffuse midline glioma	IV	1+	O	X	O
31	F	4 (D)	11.2	left frontal lobe	ganglioneuroblastoma	IV	-ve	O	O	—
32	F	8	8.2	left parietal lobe	anaplastic ganglioglioma	III	-ve	—	—	—
33	F	11	4.0	right thalamus	anaplastic astrocytoma	III	4+	O	X	O
34	M	42 (D)	5.0	left frontal lobe	anaplastic oligodendroglioma	III	2+ (n=3)	O	O	X
35	M	49	2.3	left frontal lobe	pleomorphic glial neoplasm	IV	4+ (n=3)	O	O	O

[a] Age at time of surgical procedure, in years unless under 12 months (W: week; M: month). D: deceased as of Oct 2019

[b] Largest dimension measured on presurgical MRI

[c] Consensus of two pathologist, one sample (n=1) per patient unless specified otherwise

[d] X: positive test result; O: negative test result; —: not tested

[e] Focally 4+ EGFR positive tumor cells found in one of two sample

[f] Two EGFR-SEPT14 fusion events found in the sequencing data in addition to EGFR amplification

Supplementary Figure S1. Panitumumab-IRDye800 competition assay on U251 glioma cell line. U251 cells were incubated with panitumumab-IRDye800 (5nM) and increasing concentrations of unlabeled panitumumab from **A**, 0.05nm to **G**, 5μM. **H**, Fluorescence intensity (Mean ± SEM) decreased as unlabeled panitumumab concentration increased. The half maximal inhibitory concentration (IC50) was 43.6 nM.

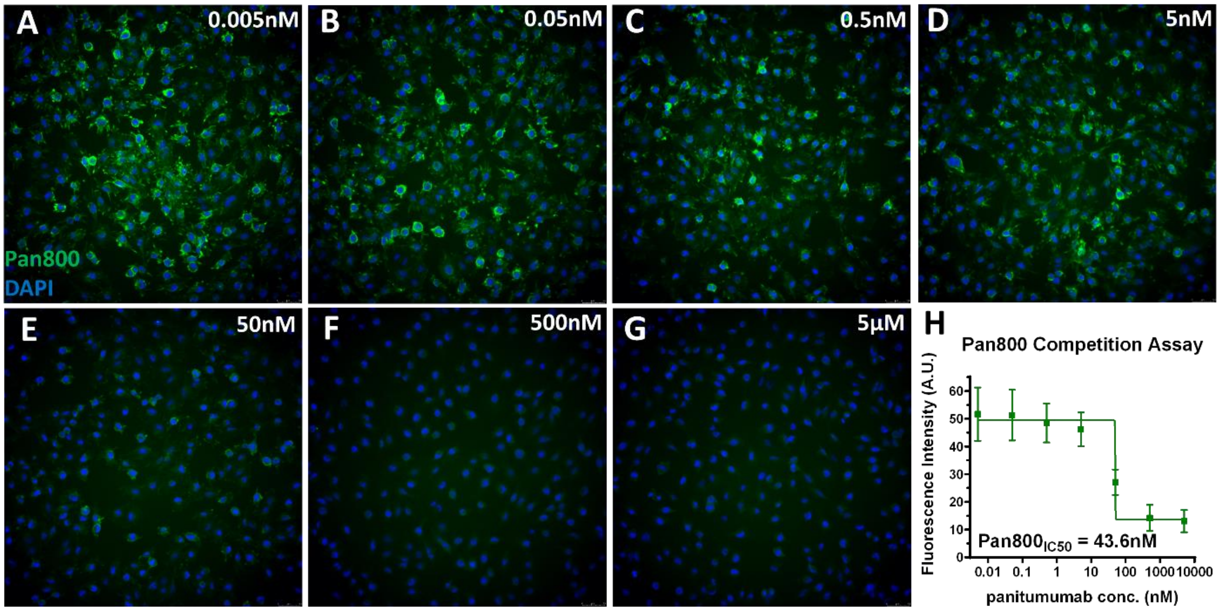

Supplementary Figure S2. Quantification algorithm segments positive immunohistochemical staining. **A**, EGFR and **B**, claudin-5 (Cldn5) immunohistochemical (IHC) staining results were segmented into positive and negative pixels according to staining intensity. *Lower panels*: a quantification mask is applied to each pixel of IHC images above with the color code of blue (negative), yellow (weakly positive), orange (medium positive) and red (strongly positive).

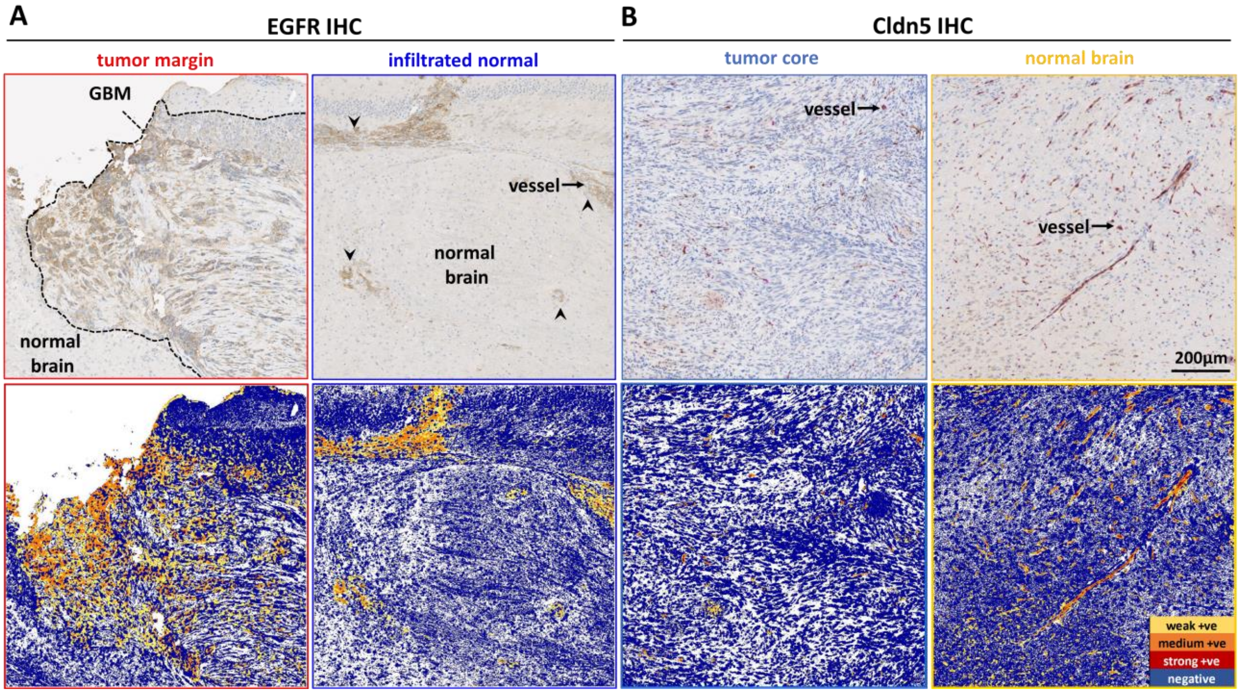

Supplement: Supplementary file 1 — Supplementary Information. [file 41598_2021_84831_MOESM1_ESM.pdf]
